# Reliability of computed tomography measurements in assessment of thigh muscle cross-sectional area and attenuation

**DOI:** 10.1186/1471-2342-10-18

**Published:** 2010-08-11

**Authors:** Sören Strandberg, Marie-Louise Wretling, Torsten Wredmark, Adel Shalabi

**Affiliations:** 1 Department of Radiology, Karolinska University Hospital, Karolinska Institutet, Stockholm, Sweden; 2 Department of Orthopaedic Surgery, Karolinska University Hospital, Karolinska Institutet, Stockholm, Sweden

## Abstract

**Background:**

Advancement in technology of computer tomography (CT) and introduction of new medical imaging softwares enables easy and rapid assessment of muscle cross-sectional area (CSA) and attenuation. Before using these techniques in clinical studies there is a need for evaluation of the reliability of the measurements. The purpose of the study was to evaluate the inter- and intra-observer reliability of ImageJ in measuring thigh muscles CSA and attenuation in patients with anterior cruciate ligament (ACL) injury by computer tomography.

**Methods:**

31 patients from an ongoing study of rehabilitation and muscle atrophy after ACL reconstruction were included in the study. Axial CT images with slice thickness of 10 mm at the level of 150 mm above the knee joint were analyzed by two investigators independently at two times with a minimum of 3 weeks between the two readings using NIH ImageJ. CSA and the mean attenuation of individual thigh muscles were analyzed for both legs.

**Results:**

Mean CSA and mean attenuation values were in good agreement both when comparing the two observers and the two replicates. The inter- and intraclass correlation (ICC) was generally very high with values from 0.98 to 1.00 for all comparisons except for the area of semimembranosus. All the ICC values were significant (p < 0,001). Pearson correlation coefficients were also generally very high with values from 0.98 to 1.00 for all comparisons except for the area of semimembranosus (0.95 for intraobserver and 0.92 for interobserver).

**Conclusion:**

This study has presented ImageJ as a method to monitor and evaluate CSA and attenuation of different muscles in the thigh using CT-imaging. The method shows an overall excellent reliability with respect to both observer and replicate.

## Background

Injury of the anterior cruciate ligament (ACL) is a common injury especially in younger people involved in different kinds of sport activity [1,2]. It is well known that atrophy of the quadriceps femoris muscle is linked to disuse both pre- as well as postsurgical in patients with ACL and/or meniscal damage. Several strategies for treatment, both conservative and surgical, have been used [3]. Different surgical strategies have been applied and there is still an ongoing development of the surgical techniques [2,4]. For evaluation of outcome and follow-up there is a need for a reliable method for measuring muscle size. There is also a need for evaluating of atrophy of the different muscles of the thigh especially as some of the techniques involve the use of grafts either from gracilis and/or semitendinosus tendons or the use of Bone-Patellar Tendon-Bone (BTB) graft. Improvements in digital imaging and imaging software have made it possible to perform such measurements easily. However, it is important to ensure that there is a good reproducibility. In an ongoing study of the outcome of ACL-surgery we had the ethics committee approvement to perform CT examinations and we therefore decided to use these examinations to evaluate the reliability of one method.

Many different methods both radiological and others have been used for evaluation of body composition. A review of the different methods was published by Mattson and Thomas in 2006 [5]. The methods used to evaluate cross-sectional area (CSA) of skeletal muscle are mainly computer tomography (CT), magnetic resonance imaging (MRI) and ultrasonography (US). There are some studies comparing two or all three of these methods, some of them also correlating the measurements with anatomical studies [6-8]

Several methods have been used to measure CSA. Most of the studies have used some kind of planimetry where the borders of the muscles are manually traced. Some have used the CT-scanners' own computer to measure the area within certain limits of attenuation. Steiger et al [9] have developed an autocontouring technique which semiautomatically delineates the muscles. Lemieux et al [10] have used a technique with imaging densitometer used on X-ray films.

Since the first CT-studies there has been a rapid improvement of the CT-scanners. The image acquisition is much faster and the images have a higher spatial resolution often with a lower radiation dose. The use of new medical imaging softwares, which made it possible to measure areas within specified attenuation limits, has made it possible to exclude interspersed adipose tissue which was generally not possible when CSA was measured with planimetry. Moreover it makes it easy to record the mean attenuation, which reflects functionality of the muscle. The software used in this study, ImageJ, is a free-ware open-source medical imaging software which can run on major computer operating systems. It can be used for images stored in DICOM standard which makes it independent on the CT-scanner used. In this study CSA and attenuation for the individual muscles in the thigh were measured from CT-examinations with ImageJ, which to our knowledge has not been done before.

The aim of the study was to evaluate the inter- and intra-observer reliability of ImageJ in measuring thigh muscles CSA and attenuation in patients with ACL injury by CT.

## Methods

To evaluate the measuring method we used subjects selected from an already existent study of rehabilitation and muscle atrophy after ACL-reconstruction with semitendinosus and gracilis tendon graft. The Ethics Committee at the Karolinska Institutet approved the design of the study, and the patients gave their informed consent of the planned procedures. For our reliability study we included the first 31 examined patients (22 men and 9 women). The median age of these patients was 27 years with a range from 16 to 45 years. All the CT-examinations included in this study were performed before surgery.

Axial CT images were acquired at three levels. At the level of, as well as 50 mm and 150 mm above the knee joint with the patients in a supine position. For assessing the reproducibility it was, according to our opinion, enough to evaluate the level of 150 mm above the knee joint which is best suited for evaluation of muscle CSA of the levels examined. The scans were performed by a Philips Tomoscan SR 7000 (single slice helical CT- scanner, 100 kV and 75 mAs) for 26 patients and with a Siemens Volume Zoom (4 slice MDCT-scanner, 120 kV and 40 mAs) for 5 patients. The use of two different CT-scanners was due to change of equipment at our department during the study period. Slice thickness in all images was 10 mm. The images were saved as DICOM-images in the departments PACS-system for later analysis.

The images were analyzed by two investigators (MLW and SS) independently using NIH ImageJ version 1.38× software http://rsbweb.nih.gov/ij/ packages. All images were analyzed by both investigators at two times with a minimum of 3 weeks between the two readings.

Both the leg with the ACL-injury and the contralateral leg were analyzed. The muscles identified and measured were: quadriceps, sartorius, gracilis, semimembranosus, semitendinosus and biceps femoris. No attempt was made to separate the different parts of quadriceps (vastus medialis, vastus intermedius, vastus lateralis and rectus femoris) or the two heads of biceps femoris (caput longum and caput breve). Even when analyzing anatomical dissection in cadaver studies it is not always possible to separate the different parts of e.g. quadriceps [11]. On most of the images a small part of the muscles of the adductor group was also present but not measured.

**Figure 1:**
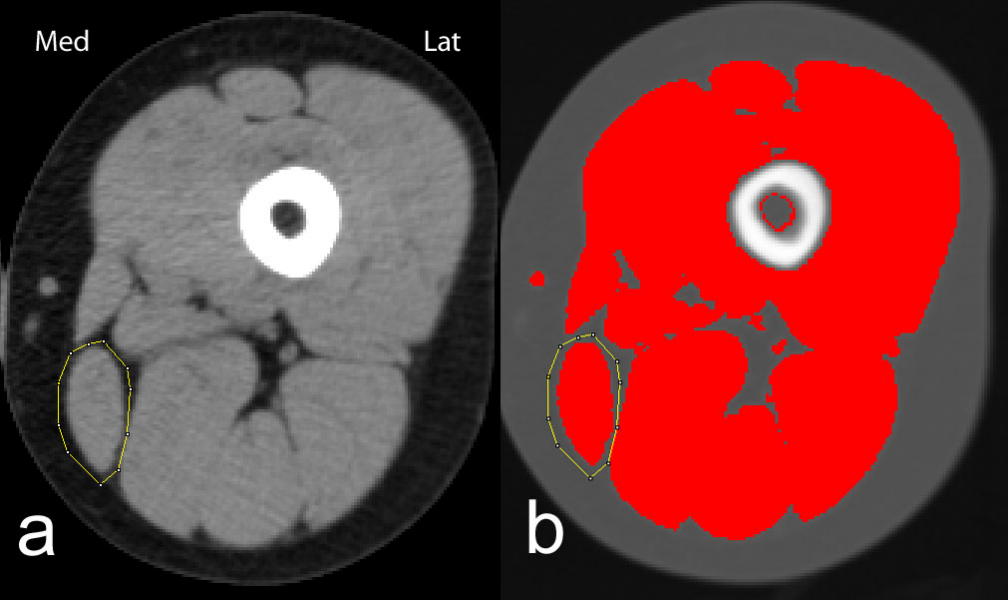
**CT image of left thigh viewed with ImageJ**. Musculus gracilis encircled. a) with level 50 HU and width 400 HU used when encircling the muscle and b) after highlighting areas with attenuation between 1 and 101 HU.

CSA of the individual muscles was measured by outlining the borders of the muscles with the polygon selection tool. This was made after adjusting the image to level 50 and window width to 400 to obtain as good visual discrimination between adipose tissue and muscle as possible. CSA was measured as the area inside the borders with attenuation values from 1 to 101 Hounsfield units (HU) (figure 1). When outlining the borders we tried to avoid nerves and vessels as they have attenuation values within the chosen limits.

**Figure 2:**
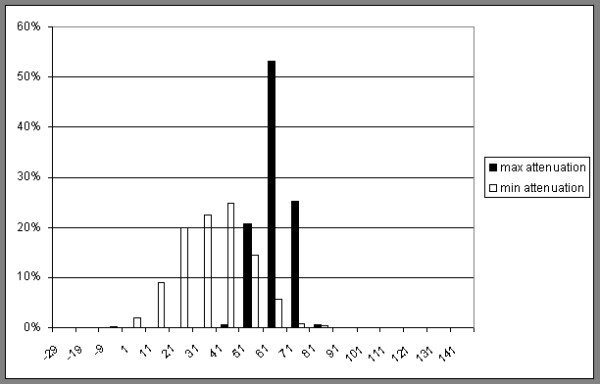
**Relative distribution of attenuation values in HU of gracilis**. Two examples chosen, with the highest and lowest mean attenuation included in the study respectively. Both are measured by drawing a line just inside the border of the muscle to avoid volume averaging at the border affecting the result.

Apart from CSA the mean attenuation of the individual muscles was also measured. For some subjects the distribution of attenuation values between -29 HU to 150 HU was also registered to test the validity of the chosen limits of attenuation (figure 2). In this case a line was drawn just inside the border of the muscle to avoid volume averaging at the border affecting attenuation values.

To improve the speed of the process we used the ability of ImageJ to use self-defined macros that reduced the amount of clicking necessary for each measurement.

### Statistical methods and data management

The test-retest reliability and the reliability based on the internal consistency was analyzed according to the method described by Bland and Altman, which yields inter- and intraclass correlation, ICC, [12,13]. The Pearson correlation coefficient was used in order to test independence between variables. In addition to that, descriptive statistics and graphical methods were used to characterize the data. All analyses were carried out by use of the SAS system, and the 5%-level of significance was considered.

## Results

Tables 1 and 2 summarize the results for the different muscles with left and right side lumped together. This means that both the healthy side and the side affected by the ACL-injury are lumped together. The differences between the healthy and affected side will be the subject of a future study.

**Table 1:** CSA of individual muscles (n = 62)

	Observer 1		Observer 2	
	**Replicate1**	**Replicate2**	**Replicate1**	**Replicate2**

Quadriceps	5787	5784	5726	5705

Sartorius	404	403	404	404

Gracilis	323	322	323	323

Semimembranosus	1420	1422	1400	1420

Semitendinosus	609	609	603	603

Biceps femoris	1739	1739	1732	1741

Mean values in mm^2^

**Table 2:** Attenuation of individual muscles (n = 62)

	Observer 1		Observer 2	
	**Replicate1**	**Replicate2**	**Replicate1**	**Replicate2**

Quadriceps	58,5	58,5	58,6	58,6

Sartorius	48,9	49,0	48,7	48,7

Gracilis	51,2	51,2	50,9	50,9

Semimembranosus	51,2	51,2	51,3	51,2

Semitendinosus	54,3	54,3	54,2	54,2

Biceps femoris	50,4	50,4	50,5	50,4

Mean values in HU

Mean CSA and mean attenuation values were in good agreement both when comparing the two observers and the two replicates but there was a difference between the different muscles with a slightly less good agreement for the area of semimembranosus.

The mean values combine men and women with different age, weight, physical condition and ACL-injury. The test-retest reliability and the reliability based on ICC were analyzed and illustrated in tables 3 and 4. The ICC was generally very high with values from 0.98 to 1.00 for all comparisons except for the area of semimembranosus. All the ICC values were significant (p < 0,001).

**Table 3:** Intraobserver reliability for area and attenuation (n = 62).

	Observer 1		Observer 2	
	**Area**	**Attenuation**	**Area**	**Attenuation**

Quadriceps	1	1	0,997	0,998

Sartorius	0,999	0,999	0,994	0,999

Gracilis	0,999	0,999	0,996	0,997

Semimembranosus	0,999	0,999	0,944	0,998

Semitendinosus	1	0,999	0,996	0,995

Biceps femoris	0,999	1	0,984	0,999

ICC comparing replicate 1 and 2

**Table 4:** Interobserver reliability for area and attenuation (n = 62).

	Replicate1		Replicate2	
	**Area**	**Attenuation**	**Area**	**Attenuation**

Quadriceps	0,993	0,997	0,994	0,998

Sartorius	0,990	0,996	0,994	0,997

Gracilis	0,992	0,992	0,995	0,992

Semimembranosus	0,970	0,997	0,922	0,997

Semitendinosus	0,985	0,994	0,988	0,995

Biceps femoris	0,985	0,998	0,992	0,998

ICC comparing observer 1 and 2

The Pearson correlation coefficients were also generally very high with values from 0.98 to 1.00 for all comparisons except for the area of semimembranosus. The reliability can also be measured as coefficient of variation (CV). The mean intra-observer CV, both observers combined, was 0.93% for CSA and 0.23% for attenuation and the mean inter-observer CV, both replicates combined, was 1.61% for CSA and 0.42% for attenuation.

**Figure 3:**
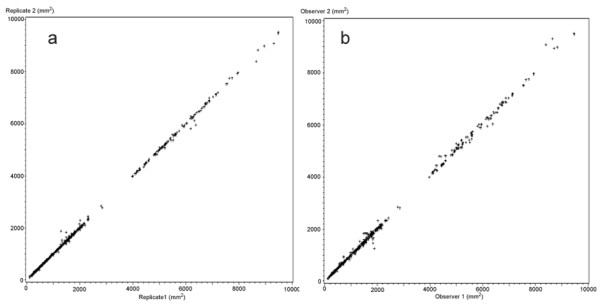
**CSA in mm**^**2 **^**for all measured muscles**. a) Intra-observer comparison, replicate 1 against replicate 2 both observers combined. (n = 744) and b) Inter-observer comparison, observer 1 against observer 2, both replicates combined (n = 744).

**Figure 4:**
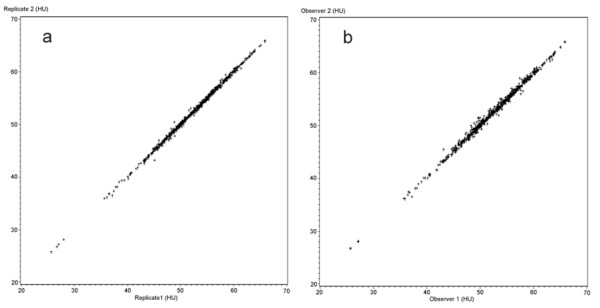
**Mean attenuation in HU for all measured muscles**. a) Intra-observer comparison, replicate 1 against replicate 2 both observers combined (n = 744) and b) Inter-observer comparison, observer 1 against observer 2, both replicates combined (n = 744).

The inter- and intraobserver reliability is also illustrated with scatterplots given in figure 3 and figure 4.

## Discussion

The main finding in the present study was the excellent overall reliability with respect to both observer and replicate in evaluation CSA and attenuation of different muscles in the thigh with the methods and equipment described above.

As noted above the method used in this study to evaluate muscle CSA and density gives results with very good reliability with respect both to inter- and intraobserver comparisons. When deciding the usefulness of the results it is also important to discuss the delimitation of muscle tissue and the interpretation of muscle density.

The definition of "skeletal muscle" in terms of HU differs. Chowdhury et al [14] used -190 to -30 HU for adipose tissue and 152 to 2500 for skeleton and manually circumscribed other organs in the range from -29 to 152. The range of -29 to 150 for skeletal muscle has later been used for example by Mitsiopoulos et al [8] and Irving et al [15].

Kelley et al [16] used -200 to -1 for adipose tissue, 0 to 100 HU for "lean tissue" and >200 for bone. They introduced the concept of "low-density lean tissue" (LDLT) for the range 1-34 HU and measured "normal-density muscle" (NDM) between 35 and 100 HU. They discussed the nature of this LDLT as it in some respects differed from normal muscle. In obese subjects there was a marked increase in LDTD but not in NDM. They discussed the possibility that this LDLT could at least partly be another tissue comprising connective tissue elements but other findings suggested that it was mostly altered skeletal muscle with higher lipid content.

Goodpaster and colleagues have used the term "low-density muscle" (LDM) for tissue with attenuation between 0 and 30 HU in several works [17-19]. Relatively higher proportion of LDM is a typical finding in obese subjects and the lower attenuation is associated with increased lipid content in muscle tissue but CT examinations cannot differ between intracellular lipid deposits and extracellular deposits as far as they has a size smaller than the pixels of the CT image [20]. Probably other factors have an effect on attenuations as well for example perfusion and difference in intra- and extracellular water content.

Reduced skeletal muscle attenuation is a typical finding in obese subjects but it is also associated with atrophy in for example rotator cuff muscles [21] and hip and knee muscles in osteoarthritis [22] where it is an independent factor apart from reduced muscular CSA, associated with reduced strength.

In this study we used attenuation from 1 to 101 HU for the measurements of the muscles. This included both LDM and NDM as defined by Goodpaster et al 1999 [17]. It was also supported by own observations where the attenuation characteristics for the two m. gracilis in our study with the highest and lowest attenuation, respectively, were compared (figure 2). In this case the attenuation values were acquired by drawing a line just inside the borders of the muscles to avoid the effect of volume averaging effect discussed later.

In obese subjects normal muscle tissue is interspersed with adipose tissue in a way that gives the muscle a marbled appearance. In lean subjects there is very little such interspersed adipose tissue [18]. The method used in this study, limiting the measured area to attenuation values between HU 1 to 101 would exclude areas of interspersed adipose tissue as far as they are larger than the pixel size but areas smaller than the pixel size would be included. At the same time the method used here, i.e. outlining the borders of the individual muscles rather roughly and then use attenuation limits for evaluation the area makes the process much faster and gives a more accurate result than manually measuring CSA.

**Figure 5:**
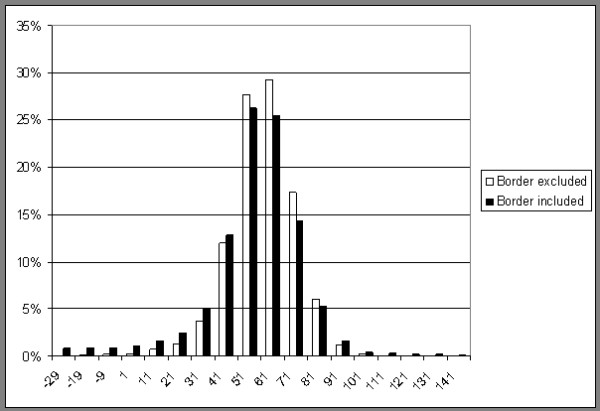
**Relative distribution of attenuation values in HU for quadriceps**. One example evaluated in two different ways by drawing a line just inside the border of the muscle (border excluded) and by drawing a line just outside the border of the muscle (border included) Relative distribution of attenuation values in HU for quadriceps One example evaluated in two different ways by drawing a line just inside the border of the muscle (border excluded) and by drawing a line just outside the border of the muscle (border included).

The concept of "volume averaging" inherent to CT imaging, i.e. if a pixel (or actually voxel) includes tissues of different attenuations, the resulting attenuation value will be an average of the different tissues, will affect the measurements. If there is a lot of interspersed adipose tissue, it can affect the resulting mean attenuation. Volume averaging also affects the outer borders of the muscles. The muscles are mostly bordered by adipose tissue or bone. The method used in this study, measuring CSA by drawing a line just outside the muscle and then using a specified range of HU to measure the area means that there will always be some pixels with attenuation values both at the lower and at the upper limit as they represent volume averaging at the borders between muscle and adipose tissue and muscle and bone respectively. To illustrate this, the same quadriceps muscle has been evaluated both in the usual way by drawing a line outside the muscle (i.e. including the borders) and by drawing a line just inside the borders of the muscle (i.e. excluding the borders). In both cases the attenuation has been evaluated from -29 HU to 150 HU and the relative distribution of attenuation values is shown in figure 5. As can be seen, the method used in this study (i.e. including the borders) adds some pixels near the lower and the upper limit but most additions are of pixels with attenuation values that will not affect CSA or the mean attenuation as they are outside the range used for muscle i.e. 1 to 101 HU. The high spatial resolution of CT-imaging today also reduces the effect of volume averaging both on attenuation and area measuring.

With lean subjects there is very little adipose tissue between the muscles which sometimes makes it difficult to delimit the individual muscles. This accounts for some of the variation of area measurements, although as shown by the very high correlation coefficients this is not a major problem. In case of semimembranosus, the main reason for the slightly bigger differences is on the other hand probably that anatomical variation sometimes makes it difficult to delimit semimembranosus from the adductor muscles that are present in some of the patients at this level of the thigh. For longitudinal studies this problem could possibly be overcome by evaluating different examinations of the same patient at the same time.

To obtain reproducible results it is also important to control the time the patient remains in supine position as the change in hydrostatic pressure when the patient moves from upright to supine position has been shown to affect CSA and attenuation [23]. We have not standardized the time in supine position which is a limitation in our study. However, the examination time is generally short. Furthermore, a recent study showed that the effect of changing position can be minimized by examining the patient within 10 min from taking the supine position [24].

Another limitation of the study is that it was designed to test the reliability of measurements from a single set of CT images. Repeated examinations might introduce errors both from technical aspects of the CT-scanner and probably more important from the repositioning of the patient. However a study published by Goodpaster et al [20] has shown these errors to be small with a coefficient of variation for the measurement of attenuation of thigh muscle to be 0.51% We also believe that the use of two different CT-scanners does not effect the results in a significant way.

Not having made repeated examinations of the same patient at the same time is a weakness of this study but it was chosen mainly to keep the radiation dose to the patients low. In a planned longitudinal study of rehabilitation and muscle atrophy after ACL-surgery the contralateral thigh will serve as a control and thus at least reduce this problem.

## Conclusion

This study has presented ImageJ as a method to monitor and evaluate CSA and attenuation of different muscles in the thigh using CT-imaging. It has proved to give excellent overall reliability with respect to both observer and replicate.

## Competing interests

The authors declare that they have no competing interests.

## Authors' contributions

SS carried out measurements, participated in statistical analysis, drafted the manuscript. MLW participated in the design of the study, coordination of the examinations, carried out measurements and in drafting of the manuscript. TW participated in the design of the study and coordination of the patients. AS participated in the design of the study, coordination of the examinations and in drafting of the manuscript. All authors read and approved the final manuscript.

## Pre-publication history

The pre-publication history for this paper can be accessed here:

http://www.biomedcentral.com/1471-2342/10/18/prepub
